# Endovenous laser ablation versus mechanochemical ablation with ClariVein^®^ in the management of superficial venous insufficiency (LAMA trial): study protocol for a randomised controlled trial

**DOI:** 10.1186/s13063-016-1548-1

**Published:** 2016-08-24

**Authors:** Clement C. M. Leung, Daniel Carradice, Tom Wallace, Ian C. Chetter

**Affiliations:** Academic Vascular Unit, Hull York Medical School, University of Hull, Hull, HU3 2JZ UK

**Keywords:** Mechanochemical ablation, ClariVein^®^, MOCA, Endovenous laser ablation, EVLA, Superficial venous insufficiency, Varicose vein, Therapy, Treatment, Outcome

## Abstract

**Background:**

Endovenous thermal techniques, such as endovenous laser ablation (EVLA), are the recommended treatment for truncal varicose veins. However, a disadvantage of thermal techniques is that it requires the administration of tumescent anaesthesia, which can be uncomfortable. Non-thermal, non-tumescent techniques, such as mechanochemical ablation (MOCA) have potential benefits. MOCA combines physical damage to endothelium using a rotating wire, with the infusion of a liquid sclerosant. Preliminary experiences with MOCA showed good results and less post-procedural pain.

**Methods/Design:**

The Laser Ablation versus Mechanochemical Ablation (LAMA) trial is a single-centre randomised controlled trial in which 140 patients will be randomly allocated to EVLA or MOCA. All patients with primary truncal superficial venous insufficiency (SVI) who meet the eligibility criteria will be invited to participate in this trial. The primary outcomes are intra-procedural pain and technical efficacy at 1 year, defined as complete occlusion of target vein segment and assessed using duplex ultrasound. Secondary outcomes are post-procedural pain, analgesia use, procedure time, clinical severity, generic and disease-specific quality of life, bruising, complications, satisfaction, cosmesis, time taken to return to daily activities and/or work, and cost-effectiveness analysis following EVLA or MOCA. Both groups will be evaluated on an intention-to-treat basis.

**Discussion:**

The aim of the LAMA trial is to establish whether MOCA is superior to the current first-line treatment, EVLA. The two main hypotheses are that MOCA may cause less initial pain and disability allowing a more acceptable treatment with an enhanced recovery. The second hypothesis is that this may come at a cost of decreased efficacy, which may lead to increased recurrence and affect longer term quality of life, increasing the requirement for secondary procedures.

**Trial registration:**

ClinicalTrials.gov identifier: NCT02627846, registered 8 December 2015

EudraCT number: 2015-000730-30

REC ref: 15/YH/0207

R&D ref: R1788

## Background

Varicose veins are a common disease worldwide, and in the United Kingdom, it affects approximately one-third of the population [1]. They are associated with symptoms causing pain and disability, soft tissue damage and venous ulcer, resulting in significant quality of life impairments [2] with consequent healthcare costs [3].

Endovenous thermal ablation (EVTA) techniques, such as endovenous laser ablation (EVLA), are now widely accepted and they are recognised as the first-line treatment for truncal varicose veins or superficial venous insufficiency (SVI) [4, 5]. This approach has been shown to allow an enhanced recovery, with less pain and disability, resulting in superior early quality of life (QoL) when compared with surgical ligation and stripping, and improved efficacy when compared with foam sclerotherapy [6–11]. However, thermal ablation techniques carry the risk of damaging perivenous soft tissue and/or nerves. Thus, patients are treated with tumescent anaesthesia (TA), which requires multiple injections in order to infiltrate around the entire length of the target vein. Our Public and Patient Involvement (PPI) group and patients report that one of the major burdens associated with endovenous thermal ablation is the discomfort during injections of TA. Some patients still experience weeks of post-procedural pain despite the use of TA [12].

Mechanochemical ablation (MOCA), using the ClariVein^®^ device (Vascular Insights, Madison, CT, United States) is a newer treatment aiming to match the efficacy of thermal ablation whilst using a gentle sclerotherapy technique, with no requirement for TA. A catheter placed within the vein deploys a rotating hollow wire which causes physical damage to the endothelium and the vein goes into spasm. At the same time, a sclerosing agent is injected through the hollow wire into the vein, which results in protein denaturation, endothelial destruction and endoluminal fibrosis [13, 14]. Since no heat is involved and TA is made redundant, thermal-related complications such as pain, haematoma, induration and nerve injury could be reduced.

In the first human safety study, 30 great saphenous veins (GSVs) with primary SVI in 29 patients were treated with MOCA using sodium tetradecyl sulphate (STS) (Sotradecol). At 6 months [15] and at 2 years [16], the technical efficacy was 97 % (29 of 30) and 96 % (27 of 28), respectively. Several early case series have reported technical efficacy, with occlusion rates varying from 87 to 97 % [17–20]. There were no serious adverse events such as pulmonary embolism (PE), deep vein thrombosis (DVT) or nerve injury observed in all previous studies. Furthermore, prospective observational cohort studies suggest MOCA was associated with significantly less procedure times, lower post-procedural pain and faster recovery than endovenous thermal ablation techniques [21, 22]. To date, no studies have been performed to compare MOCA with EVLA in the treatment of SVI. The current study has been designed to compare MOCA with EVLA in the treatment of SVI in a randomised controlled trial (RCT).

## Methods/Design

### Study objectives

The aim of this randomised clinical trial is to establish whether MOCA is superior to the current first-line treatment, such as EVLA. The two main hypotheses are that MOCA may cause less initial pain and disability, allowing a more acceptable treatment with an enhanced recovery. The second hypothesis is that this may come at a cost of decreased efficacy, which may lead to increased recurrence and affect longer term quality of life, increasing the requirement of secondary procedures.

### Study design and setting

This is a phase IV randomised clinical trial in the setting of a University Teaching Hospital, based in United Kingdom, offering tertiary vascular surgery services.

### Inclusion criteria

The inclusion criteria are: aged 18 or over; primary symptomatic SVI; reflux greater than 0.5 seconds in the saphenous veins; clinical grades C2 to C6; proposed treatment lengths of at least 10 cm; treatment with either EVLA or MOCA is technically feasible; and written informed consent.

### Exclusion criteria

The exclusion criteria are: one of the treatments is thought to be preferable by either the patient or an experienced endovenous specialist; known allergy to medications or dressings used in the treatment; known right to left circulatory shunt; evidence of acute deep vein thrombosis or complete ipsilateral occlusion; pelvic vein insufficiency; active or recent thrombophlebitis (within 6 weeks); impalpable foot pulses with the Ankle Brachial Pressure Index of less than 0.8; pregnancy or breast feeding; active malignancy; immobility; involvement in other Clinical Trials of an Investigational Medicinal Product (CTIMP) within the last 6 months; and unwell or inability to comply with the requirements for follow-up visits.

### Treatment

All varicose vein procedures were carried out under ultrasound guidance and local anaesthesia (LA) by vascular surgeons who were experienced in both techniques of endovenous ablation. No preoperative analgesia or sedation is used. Initial vein access was performed under ultrasound guidance after injection of local anaesthetic (1 % lidocaine). With the patient in the reverse Trendelenburg position, the target vein will be cannulated percutaneously under duplex ultrasound scan (DUS) at the lowest point of demonstrable reflux using a 5-Fr sheath and dilator, followed by a 0.018” guidewire. The total length of truncal vein treated will be documented.

For EVLA, the dilator and guidewire will be exchanged for a larger 0.035” wire using the Seldinger technique. The treatment sheath is then introduced and its tip accurately positioned at the saphenofemoral junction (SFJ) or saphenopopliteal junction (SPJ) (as appropriate) under DUS, aiming for a flush occlusion. The patient is then put placed in the horizontal position, and perivenous tumescent anaesthesia (TA) is infiltrated around the entire length of the target vein under DUS using a spinal needle and a pedal-operated peristaltic pump. The constituents of TA are 100 ml of 1 % lidocaine with 1:200,000 epinephrine in 900 ml of 0.9 % sodium chloride, which is buffered to pH 7.4 with 10 ml of 8.4 % sodium bicarbonate. A sterile NeverTouch Gold-Tip laser fibre is introduced and locks with the treatment sheath. Endovenous laser energy is delivered using the VenaCure 1470 nm laser generator (Angiodynamics, Waterlooville, UK) set at a continuous power delivery of 10 W.

During MOCA, the ClariVein^®^ catheter (Vascular Insights, Glasgow, UK) will be inserted, aided by the 0.018” guidewire, and its tip is placed near the SFJ and SPJ, as appropriate under DUS. The catheter is connected to a 9 V battery-motorised handle unit that controls wire rotation, and the patient is treated in a horizontal position. The wire is activated for 10 seconds in order to induce vasospasm, and the device is withdrawn with a speed of approximately 7 seconds per centimetre, while the sclerosant is continuously injected. The sclerosing agent will be sodium tetradecyl sulphate (STS), also known as STD injection and marketed as Fibrovein 3 %, 1 %, 0.5 % and 0.2 % Solution for Injection (STD Pharmaceutical Products, Hereford, UK). The concentration used will be 1.5 %, made from 2 ml ampoule of 3 % STS with equal measures of 2 ml sterile water (water for injection). Alternatively, diluting with 2 ml of 0.9 % normal saline is also satisfactory. The concentration and volume used will be clearly documented.

If required, concomitant ambulatory phlebectomy of the varicose tributaries will then be performed using the standard Oesch hook technique through 1–2 mm stab incisions. The same TA will be infiltrated around any tributaries to be treated. The phlebectomy sites will be dressed with Steri-Strip™ (3 M, St Paul, MN, USA), cotton wool, gauze and elastic compression dressings applied from foot to knee or groin, as appropriate. This will be exchanged for a thigh-length anti-thromboembolism stocking for 6 days after 24 hours. Patients will be advised to immediately mobilise within their comfort level, and to return to their normal activities and work as soon as they feel able to. Analgesia will not be routinely prescribed.

### Primary outcomes

The joint primary outcomes will assess the hypothesised advantages and disadvantages of MOCA when compared with EVLA. The first will be patient-reported intra-procedural pain measured on a standardised 100-mm visual analogue scale (VAS). The second will be technical efficacy at 1 year, with successful procedure defined as complete occlusion of the target vein segment. This will be assessed using duplex ultrasound.

### Secondary outcomes

The secondary outcomes are procedure time, post-procedural pain, analgesia use, clinical severity, generic and disease-specific quality of life (QoL), bruising, complications, satisfaction, cosmesis, time taken to return to daily activities and/or work, and cost-effectiveness analysis following EVLA or MOCA.

The procedural time is from time of starting preparation to time of finishing dressing. Post-procedural pain will be recorded on a 100-mm VAS during the first week after treatment. The type and daily dosage of any analgesia taken by patients will also be recorded in a diary for the first week. Bruising, satisfaction with treatment and cosmesis will be recorded on a separate 100-mm VAS.

Major complications include deep vein thrombosis, pulmonary embolus, stroke, loss of vision, damage to major artery, vein and/or motor nerve. Minor complications include superficial thrombophlebitis, numbness, ecchymosis, persistent tenderness, lumpiness, skin staining and wound infection.

Disease-specific QoL is evaluated using the Aberdeen Varicose Vein Questionnaire (AVVQ), the Chronic Venous disease quality of life Questionnaire (CIVIQ-20) and the VEnous INsufficiency Epidemiological and Economic Study to evaluate Quality of Life and Symptoms (VEINES-QOL/Sym). AVVQ is a validated instrument that reflects the health status impairment associated specifically with venous disease [23]. CIVIQ-20 was developed more recently and it is designed to be more patient-centred [24]. VEINES-QOL/Sym is a 26-item patient-reported disease-specific questionnaire to evaluate the QoL and symptoms across the full spectrum of conditions related to chronic venous disorders of the leg [25].

Generic QoL is assessed using the Short Form 36-item (SF-36) and EuroQol 5-domain utility index (EQ5D). The SF-36 is a multidimensional measurement of general health, which yields eight domains of functional health and well-being scores [26–28]. EQ5D is an index scale mapping three available responses to five domain questions [29], which is transformed using the UK time trade-off tariffs into a global single index scale [30]. Both have undergone extensive testing of validity and reliability, including in the context of venous insufficiency and treatment [31–33].

Two validated objective measures will be used to assess and classify the severity of disease. The first is the Clinical severity, Etiology, Anatomy and Pathophysiology (CEAP) classification, which classifies severity into six grades. The second is the Venous Clinical Severity Score (VCSS), which grades three components from 0–3 with increasing severity, and then summates them into a single score. Clinical success is the objective improvement of clinical outcome after treatment.

Post-procedural DUS will assess the treatment efficacy [34]. Initial treatment success will be defined as complete target vein occlusion at 1 and/or 6 weeks. Anything else will be regarded as a technical failure. Recanalisation will be assessed at 52 weeks and is defined as blood flow within the target vein which has been treated. This will be broken down into partial < 25 % or full ≥ 25 % of the length of the treated vein. Residual disease is regarded as any reflux which was also present at baseline, but not a target for ablation. Disease progression will be defined as any reflux within a vein which was not present on baseline assessment. In the presence of clinical recurrence, DUS will be used to map out the pattern of the recurrence as this may give insight into techniques to avoid further recurrence and aid understanding of how recurrence comes about after these novel treatments. Post-procedural DUS will also look for evidence of complications such as heat-induced thrombosis, deep vein thrombosis, haematoma and superficial thrombophlebitis.

### Trial timescales

**Fig. 1:**
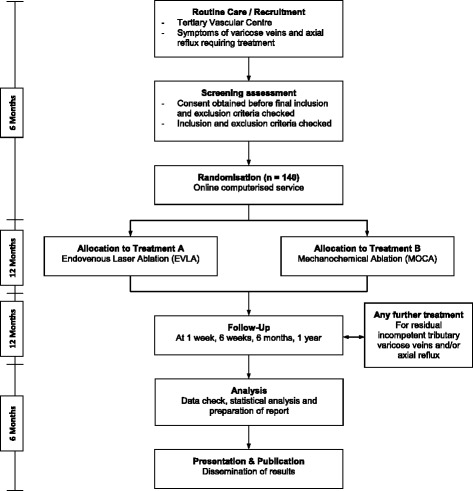
Study flow chart. The total target sample size is 140, which will be randomised to endovenous laser ablation (*n* = 70) or mechanochemical ablation (*n* = 70)

At the current rate of patients presenting to the unit, relatively wide inclusion criteria and experience from previous trials of this nature, it is anticipated that the recruitment and treatment phase would be complete within approximately 18 months. The primary endpoint is measured at 1 year and therefore completion of data collection is anticipated in approximately 30 months (Fig. 1).

### Sample size calculation

The power calculation is based upon the joint primary endpoints with 90 % power and 5 % significance. A published comparison of MOCA and radiofrequency thermal ablation found a reduction in intra-procedural pain from 35 mm to 19 mm on a 100-mm visual analogue scale (VAS) with a standard deviation of 20 mm [35]. This gives a required sample size of 33 patients per group or 73 in the trial including 10 % loss to follow-up. This difference is comparable with differences in patient-reported pain VAS, which was previously found to be associated with a difference in physical domains of quality of life and associated with changes in recovery time [8], and therefore can be judged to be clinically significant. Previous comparisons of radiofrequency ablation (RFA) with EVLA have suggested that EVLA is associated with more post-procedural pain [12, 36–38]. However older laser delivery technology (bare-tip fibres), shorter wavelength of laser (810–980 nm), additional treatment to truncal varicose vein with the allocated treatment at the same sitting, and general anaesthesia (GA) were involved; thus their findings may not be applicable today. Furthermore, no study has shown any significant benefit in quality of life relating to the reported differences.

The same study [35] reported complete target vein occlusion in 83 % of patients following MOCA at 1 month. Our previous RCT found target vein occlusion in 99 % at 1 year following EVLA [7]. A difference of this magnitude is likely to be clinically significant and have implications towards the long-term durability of the procedure, affecting effectiveness and cost-effectiveness. The required sample size to detect such a difference, if it exists, would be 62 per group or 137 including 10 % loss to follow-up. Taking this into consideration, the target sample size is 140.

### Recruitment

Each patient referred to the vascular surgery services with symptomatic SVI is assessed. Patients who potentially meet the inclusion criteria will be made aware of this research study and provided with the appropriate information, including the Patient Information Sheet. Patients expressing an interest in participation will be offered an appointment for a screening visit with a study investigator. At the screening appointment, the medical history and examination will be reviewed, followed by a detailed duplex ultrasound examination according to a set protocol based upon international consensus [39, 40]. If the potential participant meets the required inclusion criteria without any exclusion criteria, subsequent discussion of the study will take place in full (Fig. 1). If the potential participant meets the inclusion criteria for the study and is willing and able to proceed to enrolment in the trial, they will then be consented using a standardised Patient Consent Form. The Co-Investigators and Principle Investigator will obtain informed consent. Here the potential participant will be fully briefed on the trial process, the treatments, follow-up, time commitments and that this will be more detailed than regular follow-up within the non-trial setting.

To ensure confidentiality and to adhere to the Caldicott and Data Protection guidance, the participant will be assigned a unique study number for identification purposes; this will not allow identification of the study arm or any demographic information. No information identifying individuals including the study ID number will be made available to anyone outside the research group. A letter will be sent to the participant’s General Practitioner to inform them of their enrolment into the study and its details.

### Randomisation

Consented participants will be allocated to one of the two parallel treatments groups by equal randomisation (Fig. 1), which will be conducted using an online computerised service (Sealed Envelope, London, UK).

### Blinding

Due to the nature of the procedures involved it will not be possible to blind the participant or clinical team as to which group the participant is allocated. Where possible, assessor- reported outcomes will be performed by an independent assessor who is blinded to the treatment allocation. Bias in other outcomes will be limited by the use of predetermined standardised objective measurements, standardised protocols, and the extensive use of patient-reported outcomes measures.

### Data collection

Data for all outcomes from each participant’s visits will be assimilated into the participants’ unique case report form (CRF) and anonymised into a Microsoft Access database to allow further analyses, and monitored by the Research & Development (R&D) department.

### Study visits

Baseline measurements will be collected from all participants once consent is obtained and prior to randomisation. Study measurements will be taken on the day of treatment and on follow-up at 1 week, 6 weeks, 6 months and 1 year. Clinical assessment, duplex ultrasound and questionnaires are completed at these follow-up time points (Table 1).

**Table 1:** Schedule of assessments

Visits	1	2	3	4	5	6
Procedures	Screening, eligibility, baseline assessment and randomisation	Treatment	1-week follow-up	6-week follow-up	6-month follow-up	1-year follow-up
Medication history	X	X	X	X	X	X
Medications	X	X	X	X	X	X
Physical examination	X	X	X	X	X	X
NS-SEC	X					
Employment status	X					
Informed consent	X					
CEAP	X		X	X	X	X
VCSS	X		X	X	X	X
AVVQ	X		X	X	X	X
CIVIQ-20	X		X	X	X	X
VEINES-QOL/Sym	X		X	X	X	X
SF-36	X		X	X	X	X
EQ5D	X		X	X	X	X
DUS	X		X	X	X	X
Pain VAS		X	X	X	X	X
Analgesia diary		X	X	X	X	X
Satisfaction VAS			X	X	X	X
Cosmesis VAS			X	X	X	X
Recovery time			X	X	X	X
Complications			X	X	X	X

*NS-SEC* National Statistics Socio-Economic Classification, *CEAP* Clinical severity, Etiology, Anatomy and Pathophysiology, *VCSS* Venous Clinical Severity Score, *AVVQ* Aberdeen Varicose Vein Questionnaire, *CIVIQ-20* Chronic Venous Insufficiency Questionnaire, *VEINES-QOL/Sym* VEnous INsufficiency Epidemiological and Economic Study to evaluate Quality of Life and Symptoms, *SF-36* Short Form 36-item, *EQ5D* EuroQol 5-domain utility index, DUS duplex ultrasound, *VAS* visual analogue scale

### Statistical analysis

Results will be evaluated based on an intention-to-treat analysis. Continuous data will first be tested for normality. Normally distributed data will be presented as mean and standard deviation, and hypothesis testing performed with paired and unpaired *t* tests, using two-sided significance tests with a 5 % significance threshold. If the data is not normally distributed, median and interquartile range values will be presented, with analysis using the Mann-Whitney *U* test for unrelated samples and Wilcoxon signed rank test for paired data. Categorical data will be analysed by means of *X*^2^ test or, if necessary, Fisher’s exact test. Occlusion rates will be presented as Kaplan-Meier curves, including censoring.

### Monitoring, safety and quality control

This study will be monitored in accordance with the Research & Development (R&D) Department’s standard operating procedures to ensure compliance with the International Conference on Harmonisation, Good Clinical Practice and the Research Governance Framework 2005.

Adverse events (AE) are defined as any untoward medical occurrence in a subject whom a medicinal product has been administered, or a procedure performed, as part of a research study, including occurrences which are not necessarily caused by or related to that investigational medicinal product (IMP). Serious adverse event (SAE) is any event if it results in death, is life-threatening, requires hospitalisation or prolongation of existing hospitalisation, results in significant or persisting disability or incapacity, and is a congenital anomaly or birth defect. Adverse reaction (AR) is any untoward or unintended response in a subjection to an IMP. Serious adverse reaction (SAR) is a serious event which is suspected (possibly, probably or definitely) to be related to an IMP and expected for the IMP. Suspected unexpected serious adverse reaction (SUSAR) is a serious event which is suspected (possibly, probably or definitely) to be related to an IMP and unexpected for the IMP, i.e. not previously documented in any of the IMP information (SmPC) or protocol.

The AE reporting period for this trial begins as soon as patients have consented to the trial and ends 30 days after the patient’s treatment visit. The health status of subjects will be checked at each study visit. The investigator will record all directly observed AE and all AE spontaneously reported by the trial subject. A pre-existing condition is a disorder present prior to the patient entering the trial and does not need to be reported as an AE unless the condition worsens or episodes increase in frequency during the AE reporting period. All AE will be followed up by investigators until the event has resolved or a decision has been taken for no further follow-up. All AE (serious and non-serious) will be recorded by the investigator in the case report forms. A description of the event, including start date, end date, action taken and the outcome will be provided.

Investigators will notify the R&D Department of any SAE within 24 hours of becoming aware of the event. The R&D Department will report fatal or life-threatening SUSARs to the Medicines and Healthcare products Regulatory Agency (MHRA) within 7 days and follow-up information in a further 8 days. The R&D Department will send all other SUSAR reports to the MHRA within a maximum of 15 days. The investigator will repot fatal or life-threatening SUSARs to the Research Ethics Committee (REC) within 7 days and follow-up information within a further 8 days. The investigator will send all other SUSAR reports to the REC within a maximum of 15 days. The investigators will submit a Development Safety Update Report (DSUR) to the MHRA 12 months after the date of the MHRA clinical trial authorisation and thereafter until the end of the study according to the MHRA website.

Participants have access to information on complaints procedure and for obtaining compensation and treatment following harm through negligence or non-negligence as a direct result of participating in the trial.

The end of trial is defined as the last visit of the last subject completing their 1-year follow-up assessment. In accordance with Trust policy all personal and/or sensitive personal data (as defined by the Data Protection Act 1998) will be securely destroyed at the conclusion of the research. Non-identifiable data and other records not containing person-identifiable data may be retained for a longer period at the discretion of the Principle Investigator. These will be stored appropriately to ensure its integrity, confidentiality and accessibility.

## Discussion

Endovenous techniques have revolutionised the treatment of truncal varicose veins, and endovenous thermal ablation has become the recommended first-line treatment method [4], achieving occlusion rates of greater than 90 % [11]. However, the search for the optimum treatment method is still ongoing and recent emphasis has focused on improving outcomes such as intra- and post-procedural pain, and reducing thermal-related injury and complications. A potential solution to the problems raised by endovenous thermal ablation is the use of newer non-thermal and non-tumescent anaesthesia treatment methods such as MOCA.

The aim of the present randomised clinical trial is twofold. The first hypothesis is that MOCA may cause less initial pain and disability, allowing a more acceptable treatment with an enhanced recovery. The second hypothesis is that this may come at a cost of decreased efficacy, which may lead to increased recurrence and affect longer term QoL, increasing the requirement for secondary procedures. In order to have sufficient power, the trial was designed using a non-inferiority principle.

In conclusion, the LAMA trial is a randomised controlled trial that aims for reduction in intra- and post-procedural pain after MOCA compared with EVLA, with a similar clinical success and technical efficacy.

## Trial status

The LAMA trial began recruitment of participants in June 2015. By the end of December 2015, 75 patients had provided written informed consent and were subsequently randomised to either treatment.

## Abbreviations

AASV: Anterior accessory saphenous vein
AE: Adverse event
AR: Adverse reaction
AVVQ: Aberdeen Varicose Vein Questionnaire
CEAP: Clinical severity, Etiology, Anatomy, Pathophysiology
CIVIQ-20: Chronic Venous disease quality-of-life Questionnaire
CRF: Case report form
CTIMP: Clinical Trial of an Investigational Medicinal Product
DSUR: Development Safety Update Report
DUS: Duplex ultrasound scan
DVT: Deep vein thrombosis
EQ5D: EuroQol 5-domain utility index
EVLA: Endovenous laser ablation
EVTA: Endovenous thermal ablation
GA: General anaesthesia
GSV: Great saphenous vein
IMP: Investigational medicinal product
LA: Local anaesthesia
MHRA: Medicines and Healthcare products Regulatory Agency
MOCA: Mechanochemical ablation
NHS: National Health Service
PE: Pulmonary embolism
QoL: Quality of life
R&D: Research and Development
RCT: Randomised clinical trial
REC: Research Ethics Committee
RFA: Radiofrequency ablation
SAE: Serious adverse event
SAR: Serious adverse reaction
SF36: Short Form 36-item
SFJ: Saphenofemoral junction
SmPC: Summary of product characteristics
SPJ: Saphenopopliteal junction
STS: Sodium tetradecyl sulphate
SUSAR: Serious unexpected serious adverse reaction
SVI: Superficial venous insufficiency
TA: Tumescent anaesthesia
VAS: Visual analogue scale
VCSS: Venous Clinical Severity Score
VEINES-QOL/Sym: VEnous INsufficiency Epidemiological and Economic Study to evaluate Quality of Life and Symptoms
